# The Heritability of Mating Behaviour in a Fly and Its Plasticity in Response to the Threat of Sperm Competition

**DOI:** 10.1371/journal.pone.0090236

**Published:** 2014-02-25

**Authors:** Amanda Bretman, Anne Lizé, Craig A. Walling, Tom A. R. Price

**Affiliations:** 1 School of Biological Sciences, University of Leeds, Leeds, United Kingdom; 2 Institute of Integrative Biology, University of Liverpool, Liverpool, United Kingdom; 3 School of Biological Sciences, University of Edinburgh, Edinburgh, United Kingdom; Fred Hutchinson Cancer Research Center, United States of America

## Abstract

Phenotypic plasticity is a key mechanism by which animals can cope with rapidly changeable environments, but the evolutionary lability of such plasticity remains unclear. The socio-sexual environment can fluctuate very rapidly, affecting both the frequency of mating opportunities and the level of competition males may face. Males of many species show plastic behavioural responses to changes in social environment, in particular the presence of rival males. For example, *Drosophila pseudoobscura* males respond to rivals by extending mating duration and increasing ejaculate size. Whilst such responses are predicted to be adaptive, the extent to which the magnitude of response is heritable, and hence selectable, is unknown. We investigated this using isofemale lines of the fruit fly *D. pseudoobscura*, estimating heritability of mating duration in males exposed or not to a rival, and any genetic basis to the change in this trait between these environments (i.e. degree of plasticity). The two populations differed in population sex ratio, and the presence of a sex ratio distorting selfish chromosome. We find that mating duration is heritable, but no evidence of population differences. We find no significant heritability of plasticity in mating duration in one population, but borderline significant heritability of plasticity in the second. This difference between populations might be related to the presence of the sex ratio distorting selfish gene in the latter population, but this will require investigation in additional populations to draw any conclusions. We suggest that there is scope for selection to produce an evolutionary response in the plasticity of mating duration in response to rivals in *D. pseudoobscura*, at least in some populations.

## Introduction

Phenotypic plasticity, the ability of a genotype to exhibit a range of phenotypes depending on the environment, is widespread and a fundamental component of fitness [1], [2]. Plasticity in behaviour is of particular significance to animals as it is predicted to be rapidly, even limitlessly, reversible and inexpensive [3], [4], which is of critical importance if the environment is rapidly changeable. Such behaviours provide immediate responses to environmental change well before genetic adaptation can take place [5]. However, how such plasticity evolves is still debated, that is whether it is a by-product of directional selection on mean trait values or on the reaction norm of plasticity itself [4], [6]. In addition, selection on variation in plasticity has received very little attention [7], [8]. In order to investigate how behavioural plasticity evolves, the first step is to demonstrate that behavioural plasticity is heritable and therefore has a genetic component upon which selection can act.

Male reproductive success depends on the ability to compete for matings (pre-copulatory sexual selection) and, if females mate multiply, also for fertilisations after mating (sperm competition *sensu* [9]). The level of mating competition a male may face is not constant. The social environment can change very rapidly [10], altering encounter rate and sex ratio which influence both the number of mating opportunities and amount of potential competition [9], [11]. Males are expected to respond adaptively to this fluctuating environment, trading off investment between current and future mating opportunities [12], [13]. Males of many taxa exhibit sophisticated plastic responses to changes in the social environment, through physiological processes such as strategic ejaculate allocation [14] and also through plastic behaviour [11]. Male success in both pre- and post-copulation arenas has been found to be heritable in a variety of species. Fathers successful in gaining matings have more successful sons [15], and various traits linked to sperm competitive ability are also heritable [16], [17]. However, these traits are also likely to show genotype by environment (G×E) interactions, which have been proposed as a general resolution to the paradox of maintenance of genetic variation under strong directional selection [18]–[20]. Despite the prediction that responses to rivals should be adaptive, the fitness consequences of such responses are very rarely measured [11], and the extent to which such plastic responses are heritable has not been investigated.

Recent work in *Drosophila* has provided the first direct evidence that such plastic responses to changes in potential competition do indeed increase male fitness. In *D. melanogaster*, males use a complex range of cues to assess the presence of rivals [11], and after exposure to a rival subsequently mate for longer [21]. This has significant fitness benefits, with males that have been exposed to rivals gaining more offspring, reducing female willingness to remate, and achieving higher paternity share as either the first or second mate [21]. These effects are at least in part mediated through increased accessory gland protein (Acp) transfer [22]. A similar increase in copulation duration and offspring production has been found in *D. pseudoobscura* [23] and *D. montana* [24]. Recently, the response to rivals of increased mating duration has been shown in three more species: *D. subobscura*, *D. acanthoptera* and *D. nannoptera*, illustrating that behavioural responses to rivals are widespread in this genus [25]. However, female *D. subobscura* [26] and *D. acanthoptera* [25] remate extremely infrequently, suggesting males may not benefit by suppressing remating or increasing sperm competitive success in these species. Therefore, although adaptive explanations have been suggested for these responses [11], [21], there is currently still some debate and it is still possible that these changes in behaviour are non-adaptive. For example, when exposed to rival males, males might be attacked, harassed, or excluded from feeding, leading to a reduction in their physical state. Thus, the longer copulation durations might then be required for males exposed to rivals to achieve the same amount of sperm/Acp transfer as those that are not exposed to rivals. Alternatively females might respond differently to males that have been kept with rivals, resulting in an increase in mating duration. An improved understanding of the genetic basis of mating duration and its response to rivals would help to clarify the evolutionary potential of this trait.

Here we investigate whether there is heritable variation in male response to rivals in the fruit fly *D. pseudoobscura*. The species harbours a sex ratio distorting selfish genetic element (referred to as *sex-ratio*, or *SR*), which can create biased sex ratios in natural populations, and its prevalence can fluctuate over the course of a year [27]. Hence environmental and genetic factors are likely to drive rapid local fluctuations in the natural social environment of *D. pseudoobscura*. As mentioned above, males of this species show a similar response to rivals as *D. melanogaster*, increasing copulation duration when exposed to a rival for four days prior to mating [23]. This increase in copulation duration leads to increased offspring production by the females mated to males that have been exposed to rivals [23]. Furthermore, these males show a sophisticated plastic response in ejaculate allocation [23], increasing the transfer of fertile eusperm but not infertile parasperm. Parasperm are thought to protect the eusperm from the harsh environment of the female reproductive tract, increasing eusperm longevity [28]. The evidence therefore suggests that these behavioural and physiological responses are adaptive. We now test whether this plastic behavioural response has a genetic basis by examining the mating duration of lines of genetically identical males exposed or not exposed to a rival. We examine this only in lines that do not carry *SR*, as the *SR* chromosome is itself likely to modify male behaviour. Instead we focus on how non-*SR* males respond to the risk of sperm competition. We also test the hypothesis that populations with different prevalence of the sex ratio distorter, hence different propensity for variation in sex ratio, will show a difference in their plasticity. Specifically, that a population with low levels of *SR* will show lower plasticity, or difference in mating duration, between isolines.

## Methods

### Fly stocks

We collected flies using standard banana baits [29] from two sites in the USA, Lewistown (109°16′53″W, 47°04′47″N), Montana, and Show Low (110°07′37″W, 34°07′37″N), Arizona, in May 2008. The frequency of *SR* in each population was determined by mating 100 wild caught males to stock females. Males which produced broods that were more than 95% female were assigned as *SR* males [30]. We found no *SR* males in Lewiston, whereas 11% carried *SR* in Show Low, and previous work confirms that *SR* is found at less than 1% frequency in the Lewiston population, whereas in Show Low population it is found at 10–20% prevalence [31]. We inbred the offspring of wild caught females to create isofemale lines (hereafter isoline). For this we used lines that did not carry *SR*, as revealed both by genotyping with a PCR marker for *SR* [see methods in 32] and examination of the sex ratio of broods fathered by males from the isoline. Isolines maintain genetic diversity and prevent evolution and adaptation to the laboratory by reducing heterozygosity [33]. Briefly, we allowed each wild caught female to oviposit in a vial of standard *Drosophila* food [29]. We collected a virgin son and daughter on eclosion and placed them in a new vial to mate and produce offspring. This inbreeding limits each locus to a maximum of four alleles in that isofemale line. We continued single son and daughter matings for two more generations to reduce genetic diversity. From this point on we maintained each isoline as a small group of siblings (<12 in any generation) to increase inbreeding and reduce heterozygosity. We produced a new generation for each isoline every month. We also produced an outbred stock population by combining offspring from all the isolines. This was maintained as a large outbred population, with one generation per month. The experiments described below were carried out in July 2011 (after 35 generations in the laboratory), by which point the isolines are expected to have been almost completely homozygous, and individuals from the same isoline are expected to be genetically almost identical. All flies were maintained at 23°C, at which all experiments took place.

### Testing for genetic variation and G×E in mating duration

For this experiment we used 13 isolines from Lewiston and 14 from Show Low. We collected males from each isoline within 18 h of eclosion to ensure virginity [34]. We also collected virgin males from the stock population. Stock males to be used as rivals were marked by cutting 50% of one wing off under CO_2_ anaesthesia at 24 hours old, which does not affect the response to these males by focal males at 4 days old [35] (T. Price pers. comm. for *D. pseudoobscura*). Isoline males were randomly allocated to “exposed to rivals” (R) or “not exposed to rivals” (NR) treatments. R males were placed in a vial with a marked stock male, whereas NR males were kept singly in a vial. Flies were 5–6 days old at the time of mating, at which age both sexes are fully sexually mature [36], [37]. Hence males were maintained in their treatment condition (i.e. R, NR) for 4–5 days prior to mating allowing ample time for the response to rivals to develop [38]. We also collected virgin females from the stock population. We kept these females in groups of 15–20 per vial. The day before mating, females were moved to mating vials supplemented with live yeast granules. At mating, males were placed singly into a female vial. We used aspiration to move all flies, not anaesthesia as this can alter copulation behaviour [39]. Introduction time, start and end of mating times were noted. Observation of copulations was blinded by one researcher transferring males from their treatment vial to the vial containing the female while out of view of the two observers. As the vials containing the females were labelled only with a random number, the two observers did not know the isoline or treatment of any copulating flies. Pairs were given 3 h to mate. 51 pairs failed to mate. 457 pairs did mate, with a mean of 15 pairs mating per isoline (range 11–20).

### Estimating genetic variation

Genetic variation and genotype by (rival) environment interactions in mating duration were tested by fitting standard linear mixed effects models in ASReml [40]. We fitted models of the form:
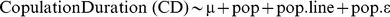
(1)


Where µ is the intercept, pop is a fixed effect of population of origin of the lines, pop.line is the random effect of line nested within population and pop.ε is the random error term nested within population. Genetic variation is estimated from the variance in mating duration that is explained by pop.line. This model was run separately on data from each environment (no rival and rival present) to provide estimates of the total genetic variance in each environment. Broad sense heritability for mating duration in each environment was calculated as the variance due to pop.line expressed as a proportion of the sum of the variance due to pop.line and pop.ε.

To test for environment specific genetic variation and differences in the response to environments across populations (G×ssE) we fit a model of the form:
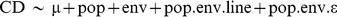
(2)


Where terms are as above except env represents a fixed effect of environment, and population and environment specific line variances and genetic covariances between lines represented in multiple environments are estimated. To test for genotype by environment interactions, this model was compared to models where genetic correlations between copulation duration in different environments in the two populations were fixed to one and where genetic variances across environments and populations were constrained to be equal. This tests the hypothesis that the genetic correlation between copulation duration in the two environments in the two populations is 1 and that the genetic variances in the two environments in the two populations are equal. Genetic correlations of less than one or unequal genetic variances between environment indicate significant G×E for this trait. Model comparisons were made using LogLikelihood ratio tests. Traditionally, significance has been based on an assumption that twice the difference in LogLikelihoods of the models is chi-squared (χ^2^) distributed with the number of degrees of freedom equal to the difference between the models in the number of parameters estimated. However, it has recently been highlighted that this approach is over-conservative and that the actual distribution is a mixture of χ^2^ distributions with different degrees of freedom [41]. In practice, for the particular case of models differing in one (co)variance parameter, this means that a more appropriate p-value is half the p-value returned assuming one degree of freedom [41]. We therefore adopt this approach throughout this manuscript. Standard errors for heritabilities and genetic correlations are returned by ASReml.

## Results

### Response to the presence of a rival

When pooled across isolines within a population, males showed a longer copulation duration when mating after exposure to a rival (mean and standard error: Lewistown, no rival: 233.09, 7.47; Lewistown, rival: 315.04, 12.29; Show Low, no rival: 229.80, 7.84; Show Low, rival: 305.68, 9.63).

### Copulation duration when not exposed to a rival

**Figure 1:**
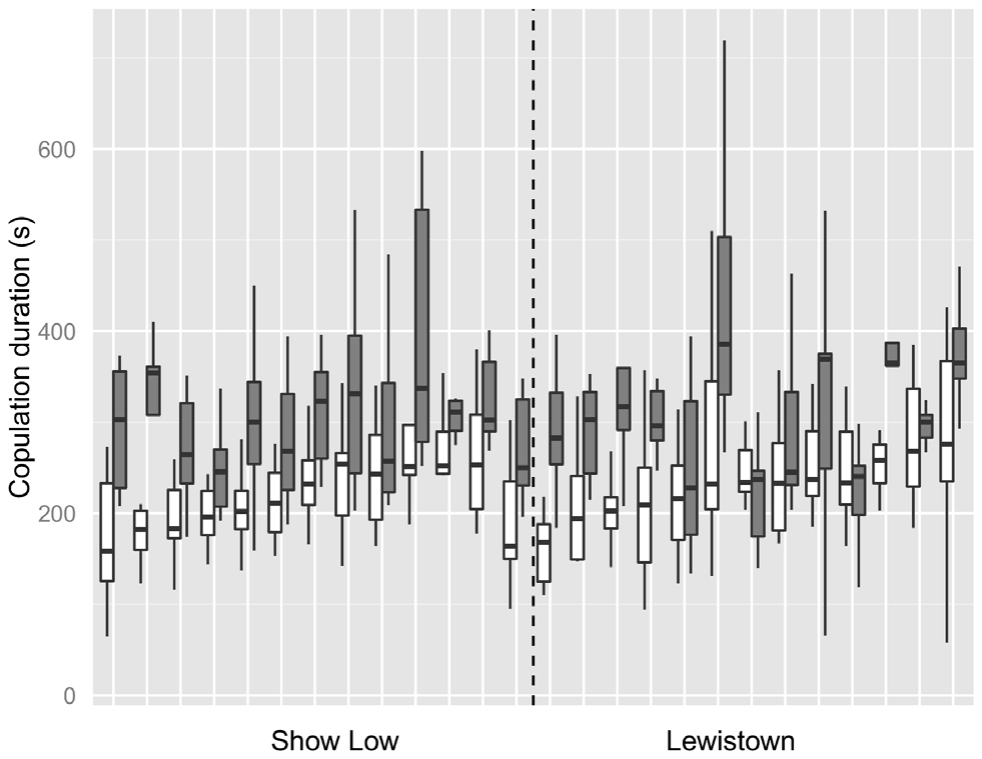
Boxplot showing the variation in copulation duration of males from 13 isofemale lines from each of two populations (Show Low and Lewistown), both when exposed to a male rival prior to copulation (filled boxes) or not exposed to rivals (hollow boxes). Median, interquartile range and total range are shown by the horizontal lines, boxes and whiskers respectively.

Univariate models of copulation duration in the absence of a rival revealed no significant difference between the amount of variation explained by line in the two populations (Log Likelihood ratio test, χ^2^_0&1df_  = 0.04, p = 0.42), showing that the genetic variation in copulation duration is equal in each population. In addition there was no significant effect of population of origin on the mean copulation duration in the absence of rivals (effect of population  = 4.00±13.75 seconds, F_1,24.4_ = 0.08, p = 0.77; see Figure 1), showing that populations did not differ overall in their copulation duration. We therefore estimated the total genetic variation and broad sense heritability of copulation duration from a model with line nested within population assuming line variances in both populations are drawn from the same distribution. Line variance and thus total genetic variation in this analysis was estimated as 484±346 giving a broad sense heritability (H^2^) estimate of 0.078±0.054, which was significantly greater than zero (χ^2^_0&1df_  = 3.32 p = 0.034).

### Copulation duration after exposure to a rival

Line variation for copulation duration was also not significantly different between the two populations when expressed in the presence of a rival (χ^2^_0&1df_ = 0.20, p = 0.33) and again there was no effect of population on mean copulation duration (estimated effect size = 7.04±21.39, F_1,24.5_ = 0.11, p = 0.75). Total genetic variation and broad sense heritability were therefore again estimated from a model assuming line variation in both populations is drawn from the same distribution. There was significant heritability in this environment (line variance = 1472.62±846.33, broad sense heritability H^2^ = 0.118±0.063, χ^2^_0&1df_  = 6.16, p = 0.0065).

### G×E in copulation duration in response to a rival

Males exposed to rivals significantly increased their copulation duration, with presence of a rival increasing the predicted mating duration by 79.81±11.85 seconds (see Figure 1; F_1,51_.9 = 45.19, p<0.001). To show that these changes in mating duration differed between isolines, and thus had a genetic basis, requires significant genotype by environment interaction (isoline × rival presence/absence). There was some marginal evidence of genotype by environment interactions in this study. The genetic correlation (r_g_) between mating duration with and without a rival present was marginally non-significantly different from 1 in Show Low (southern, high SR) (r_g_ = 0.00327±0.584, χ^2^_0&1df_  = 2.48, p = 0.058), but not significantly different from 1 in Lewistown (northern population, no SR), (r_g_ = 1.07±0.55, χ^2^_0&1df f_ = 0, p = 1). However, these correlations were not significantly different from each other (χ^2^_0&1df_  = 1.68, p = 0.097). This provides some evidence for G×E in Show Low, suggesting that the rank order of genotypes in mating duration changes when exposed or not to a rival, but that this is not the case in Lewistown, although it should be noted that the genetic correlation in Show Low is not significantly different to that in Lewistown. The total genetic variation did not differ between environments in either population (Show Low, χ^2^_0&1df_  = 0.70, p = 0.20; Lewistown, χ^2^_0&1df_  = 0.52, p = 0.24) and the genetic variance did not differ between populations (χ^2^_0&1df_  = 0.20, p = 0.32). Taken together these results provide marginal evidence of genotype by environment interaction for mating duration in Show Low, but not in Lewistown, with the genetic correlations between mating duration in different environments being marginally non-significantly different from 1 in Show Low. However, this result is technically non-significant, and so is only weak evidence of G×E effects. The data presented are for untransformed copulation duration. However, transforming copulation duration such that in each environment and population, the mean mating duration was 0 and the variance was 1, and thus controlling for any effects of scaling differences between populations and environments, gives qualitatively similar results (data not shown).

## Discussion

Males exposed to rivals increased their copulation duration, as in previous experiments [23], [25]. However, we found no significant difference between populations in mating duration *per se* either with or without exposure to a rival, or difference between mating durations in either condition (i.e. overall population degree of plasticity). Mating duration showed significant heritable variation in both conditions, with the broad sense estimate being greater for males exposed to rivals, although not significantly so. There was some variation in the degree of response to rivals amongst lines, with borderline significant variation between lines in response in the southern Show Low population, but no evidence for this in the northern Lewistown population, although this difference between populations was not significant. This provides some evidence that the degree of plasticity of mating duration is more variable in the southern Show Low population, suggesting that plasticity in copulation duration may be heritable. However, this conclusion is only supported by a borderline significant level of heritable variation, not a clearly significant result. Further examination of variation in plasticity between isolines is needed to strengthen this conclusion.

A genetic basis and heritable variation are required if a trait is to be evolutionarily labile. In this study, copulation duration was significantly heritable, both with and without the presence of rivals. However, in both cases the heritability was small (broad sense heritability: exposed to rival: 0.12; no rival: 0.07). A previous investigation of the heritability of mating duration in *D. melanogaster* found that heritability of mating duration varied between sexes, with *h^2^* = 0.23–0.46 for father-son comparisons, but *h^2^* = 0 for mother-daughter comparisons [42]–[44]. A more recent study found the opposite result, with significant dam effects on copulation duration (although they did give *H^2^* or *h^2^* estimates), but no effect of father genotype [45]. Our estimates of the heritability of mating duration are at the lower end of those generally found in other species (*h^2^* = 0.58 in *Onthophagus taurus* [17], 0.39 in *Scatophagia stercoraria* [46], 0.26–0.36 in *Callosobruchus maculatus* [47]), although these studies investigated narrow-sense heritability, and so may not be directly comparable with our broad-sense heritability estimates.

We found no evidence for overall differences in copulation duration in our two populations. To our knowledge, the only previous study to assess population differences in a response to rivals was of the soapberry bug (*Jadera haemotoloma*) in the USA [48]. Northern populations exhibit differential overwinter survival between the sexes, hence sex ratio variation, whereas southern populations do not. In a common garden experiment, males from northern populations showed plasticity in mate guarding, whereas southern males did not. This shows that such responses can be gained or lost between populations with different selection pressures. However, it must be noted that in this case the behaviour itself, mate guarding, is directly related to fitness (the longer a mate is guarded the less likely she is to remate). In our system we expect sex ratio variation to be greater in populations that have a high prevalence of the sex ratio distorter *SR* allele, due to seasonal fluctuations in its abundance and the resulting sex ratio [27]. However, we found no evidence for the presence of *SR* affecting the overall degree of response to rivals between populations, although with only two populations investigated, this is a very weak test.

The borderline significant heritability of plasticity in the Show Low population is the first evidence that suggests that the response to rival males in mating duration is heritable. Only one previous study has investigated this topic, and found no evidence for heritability of response to rivals in *D. melanogaster* [45], although this study used flies that had been maintained in a mass laboratory population for more than 20 generations, and so may have lost much of its natural genetic variation. Our borderline significant heritability of plasticity suggests that there may be significant genetic variation in the trait, and hence selection would be able to act directly on response to rivals. However, as the heritability was only borderline significant, it is premature to draw too strong a set of conclusions from this experiment. It is possible that a study with a larger sample of isolines would detect heritable variation in plasticity in both populations, or that the borderline significant result is spurious. Nevertheless, if the borderline significant heritability of plasticity in Show Low, but not Lewistown, is true, then why might there be a difference between the heritabilities in these two populations? Both populations occur in large areas of suitable forest habitat, and are likely to have very large population sizes. *D. pseudoobscura* mate at dawn and dusk, and adjust activity periods to times of suitable temperature and humidity, so despite the latitudinal difference between the populations, matings in both probably occur at similar temperatures [49]. However, the X chromosome meiotic driver SR is almost completely absent in Lewistown, never being found at higher than 1% frequency (T. Price, Pers. Obs.). The 10–20% frequency of X chromosome meiotic drive in Show Low is likely to result in female biased sex ratios, with some males likely to experience strongly female biased local sex ratios in areas where most eclosing flies are descended from a small number of SR mated females. It is possible that if populations are female biased there is less competition between males, and this may relax selection on the response to rivals and allow the maintenance of genetic variation for the response to rivals.

The lack of conclusive evidence for the heritability of plasticity in this study is possibly due to the difficulties of detecting such heritability. Phenotypic plasticity is accepted to have a genetic basis, but this is rarely quantified [8]. Nevertheless, in general, studies of plasticity in morphological traits find higher heritability of the trait value than of the plasticity of the trait [2]. Heritability of behavioural plasticity has been measured in other contexts, for example *Daphnia* swimming behaviour under differing predation and starvation environments [50] and exploration-acclimation behaviour in three-spined sticklebacks (*Gasterosteus aculeatus*) [51]. Plasticity in laying date in response to temperature has been investigated in birds (e.g. collared flycatchers *Ficedula albicollis* [52], common gulls *Larus canus* [53] and great tits *Parus major* [54]), but significant heritability in level of plasticity has only been found in great tits. Brommer *et al.* [52] highlighted the difficulties of using data from the wild for this sort of study, as factors such as condition and context dependent selection may obscure estimates, and this approach requires that individuals were observed at least twice. In the context of responses to rivals, selection for plasticity, or the ability to exhibit such plasticity, are predicted to be affected by factors both extrinsic and intrinsic to the male, such as environmental stability or individual male condition, but have not been investigated [11]. Nevertheless, these issues should be reduced in our controlled laboratory environment, hence we suggest other reasons for lack of heritability in degree of mating duration plasticity.

Firstly we must consider the power of our design to detect heritable variation. Our number of isolines is lower, and replicates per line higher, than some other GxE studies [55], although other studies of genetic variation using inbred lines have used smaller numbers of lines [56]. Thus as with many quantitative genetic studies caution should be used in interpreting a borderline significant difference between genetic parameters, particularly between genetic correlations where estimates are generally expected to be imprecise [57]. Secondly, under strong directional selection, additive genetic variation for a trait is predicted to become rapidly exhausted [58]. In *D. pseudoobscura*, responses to rivals are beneficial in terms of number of offspring [23]. There are also benefits to responding to a rival in *D. melanogaster* [59], and the complex cue system required for this response suggests that avoiding an inappropriate response is important [60]. Of the *Drosophila* species so far tested, 6/7 respond to the presence of a rival in the same manner [21], [23]–[25], [61], even monandrous species which presumably do not face sperm competition [25]. These lines of evidence suggest that selection for the ability to be plastic is so strong in this genus that it has become fixed and is not easily reduced. Thirdly, plasticity may largely be achieved by non-heritable mechanisms. It has been suggested that non-heritable epigenetic modifications may have a large role to play in behavioural plasticity [62], [63], and theoretical models suggest that plasticity generally may derive form epigenetic [2], [64]. As yet we do not know the genomic or epigenomic basis of male responses to their competitive environment, hence this line of enquiry will prove very useful in understanding how this plasticity is achieved, maintained and evolved [65].

### Conclusions

We found evidence of heritability of mating duration, both in the presence and absence of rival males. We also found evidence suggesting genetic variation in degree of plasticity in mating duration depending on exposure to rivals in one of the two populations, although this evidence was borderline non-significant and hence very weak. However, we did not find overall significant differences in plasticity between populations that are expected to show different variation in sex ratio, suggesting that this is not a strong enough selective pressure to globally diminish or increase plastic responses to mating rivals. Nevertheless, we suggest that the extension of mating duration after exposure to rivals is probably heritable, at least in one population, and so has the potential to respond to selection.
